# Health seeking behavior for cervical cancer in Ethiopia: a qualitative study

**DOI:** 10.1186/1475-9276-11-83

**Published:** 2012-12-29

**Authors:** Zewdie Birhanu, Alemseged Abdissa, Tefera Belachew, Amare Deribew, Hailemariam Segni, Vivien Tsu, Kim Mulholland, Fiona M Russell

**Affiliations:** 1 Department of Health Education and Behavioral Sciences, Jimma University, P.O.Box 378, Jimma, Ethiopia; 2 Department of Laboratory Sciences and Pathology, Jimma University, P.O.Box 980, Jimma, Ethiopia; 3 Department of Reproductive Health, Jimma University, P.O.Box 378, Jimma, Ethiopia; 4 Department of Epidemiology, Jimma University, P.O.Box 980, Jimma, Ethiopia; 5 Department of Gynecology and Obstetrics, Jimma University, P.O.Box 378, Jimma, Ethiopia; 6 PATH, Seattle, USA; 7 London School of Hygiene and Tropical Medicine, London, UK; 8 Centre for International Child Health, Royal Children’s Hospital, Dept. of Paediatrics, University of Melbourne, Melbourne, Australia

**Keywords:** Cervical cancer, Health seeking behavior, Ethiopia

## Abstract

**Background:**

Although cervical cancer is a leading cause of cancer related morbidity and mortality among women in Ethiopia, there is lack of information regarding the perception of the community about the disease.

**Methods:**

Focus group discussions were conducted with men, women, and community leaders in the rural settings of Jimma Zone southwest Ethiopia and in the capital city, Addis Ababa. Data were captured using voice recorders, and field notes were transcribed verbatim from the local languages into English language. Key categories and thematic frameworks were identified using the health belief model as a framework, and presented in narratives using the respondents own words as an illustration.

**Results:**

Participants had very low awareness of cervical cancer. However, once the symptoms were explained, participants had a high perception of the severity of the disease. The etiology of cervical cancer was thought to be due to breaching social taboos or undertaking unacceptable behaviors. As a result, the perceived benefits of modern treatment were very low, and various barriers to seeking any type of treatment were identified, including limited awareness and access to appropriate health services. Women with cervical cancer were excluded from society and received poor emotional support. Moreover, the aforementioned factors all caused delays in seeking any health care. Traditional remedies were the most preferred treatment option for early stage of the disease. However, as most cases presented late, treatment options were ineffective, resulting in an iterative pattern of health seeking behavior and alternated between traditional remedies and modern treatment methods.

**Conclusion:**

Lack of awareness and health seeking behavior for cervical cancer was common due to misconceptions about the cause of the disease. Profound social consequences and exclusion were common. Access to services for diagnosis and treatment were poor for a variety of psycho-social, and health system reasons. Prior to the introduction or scale up of cervical cancer prevention programs, socio-cultural barriers and health service related factors that influence health seeking behavior must be addressed through appropriate community level behavior change communications.

## Background

Cervical Cancer (CC) is an important public health problem worldwide. It is the third most common cancer in women accounting for approximately 12% of all cancer cases [1]. Although data from developing countries are limited and less reliable, recent evidence has shown that CC is the second commonest cause of death due to cancer in developing countries [2]. In 2010, it was estimated that 20.9 million women were at risk of developing CC in Ethiopia and the estimated annual number of CC cases and deaths was 4,648 and 3,235, respectively. It is projected that the number of new CC cases will almost double by 2025 [3]. Facility based studies have shown that CC was the leading cause of cancer in Ethiopia [4,5] and other studies had also shown that CC accounted for 25.8% to 32% of all female malignancies [6,7]. These figures would substantially underestimate the actual number of cases given the perceived low level of awareness, cost of transport and treatment, limited access to screening and treatment services, and the lack of a national cancer registry to enumerate cases. Despite the high toll of morbidity and mortality due to CC in Ethiopia, there are no community level interventions except a few isolated screening and treatment services that have recently been established.

Understanding local perceptions of health needs, the process of health decision-making, and concerns and considerations of locals, are key components to understanding health seeking behavior for any health condition [8]. Various theories and models have been developed to help understand and explain health related behavior, and suggest ways to achieve desired behavioral change [8]. The Health Belief Model (HBM) is one of the most widely used conceptual frameworks to explain and describe health related behaviors and is used as a guiding tool for health behavior interventions [8,9]. The HBM includes four main factors that determine the initiation of a particular health behavior: perceived susceptibility to a disease, perceived severity, perceived barriers, perceived benefits, and cues to action [8]. Knowledge of all of these factors is believed to be imperative to the planning process of successful interventions and the expansion of existing health services. The aims of this qualitative study are to describe the perceptions of men, women, and community leaders regarding awareness, and treatment seeking behaviors for women with symptom or signs of CC in Ethiopia based on the theoretical framework of the HBM.

## Methods

### Study design

A qualitative descriptive study design was used to explore health seeking behavior for CC in Ethiopia. This study was primarily designed to assess the acceptability by the community of introducing Human Papillomavirus (HPV) vaccine to eligible girls. A qualitative research method was chosen to allow exploration of the meanings and perceptions of participants regarding CC, including their awareness and health seeking behavior for CC, and to understand the factors affecting decision making to seek treatment.

### Study sites

Focus Group Discussions (FGD) were conducted from November 2, 2010 to January 29, 2011, in Jimma Zone, south-west Ethiopia and the capital city, Addis Ababa. Jimma zone is comprised of 17 districts, each with primary healthcare units. The zone has a total population of 2.5 million residing both in urban and rural areas. Addis Ababa has 10 sub-cities with a population size of 2.9 million and has a large migratory population mixed with a more stable local population [10].

### Sampling method

Two districts of Jimma zone (Limmu Kossa and Shebe Sombo) were selected from the 17 districts by randomly picking names. Two “kebeles”/villages (one rural and one urban) were randomly selected from each district, and schools in the selected kebeles were included in the study. From each of the selected schools, a list of girls within the age range of 9–12 years was obtained and ten girls were randomly selected using computer generated random numbers to participate in the study to assess acceptability of the HPV vaccine. An invitation letter was sent to the parents (both mother and father) of selected girls to invite them to come to the school and participate in the study and give consent for their child’s participation. To limit group size, FGD participants were selected from the parents who came to the school on the day of the FGD, by picking names randomly from a hat. Parents who were not selected were free to leave after giving consent for their child. Similar selection strategies were employed to recruit participants in Addis Ababa. Two sub-sites (Kolfie and Arada) were randomly selected and two primary schools were randomly selected from each sub-city by picking names out of a hat. The selection of parents followed the same procedures as in Jimma.

### Data collection instruments and procedures

A previously published study was used as the basis for this study’s design which was adapted for Ethiopia [11]. This published study provided a conceptual framework to undertake formative research, summarized field experience and challenges, and outlined best practices for formative research to undertake in developing countries prior to HPV vaccine introduction [11]. An open ended questionnaire, designed to inform country-level HPV vaccine delivery strategies in developing countries, was adapted for use in this study [11]. In addition, HBM constructs were incorporated into the framework and a general discussion guide was developed and adapted to the theoretical framework of the HBM. The topics included were: the perceived susceptibility and severity of the disease, and perceived benefits and barriers of seeking treatment. In addition, items to promote discussion on etiology, mode of transmission, treatment options, attitude of the community towards women affected by CC, and prevention strategies were also included.

The framework was reviewed by experts in the field and translated into two local languages (Amharic and Afan Oromo). The instruments were pretested amongst a similar target population before applying to the actual study.

The purpose and objectives of the study were explained to the participants prior to the FGD, and confidentiality of their identities was ensured. Each FGD was conducted by two trained facilitators, who each have a Masters in Public Health and were fluent in the required local languages. They used the general discussion guide to prompt discussion and elicit further details through probes. The male FGDs were facilitated by men, and the female FGDs were facilitated by women, and were conducted in parallel. Each FGD was audiotaped with each participant’s permission. Each session lasted approximately 45–90 minutes and closed after the saturation of ideas. The FGDs were conducted at schools and kebele administration offices and there were no other attendees apart from the participants and facilitators during the discussions. Detailed hand written notes of each session were taken at the time of the discussion. Participants were encouraged to speak and express their ideas freely and describe their experience with cases of CC.

### Data analysis

Data from the FGDs were captured using voice recorders, and each day field notes were transcribed verbatim into the English language by FGD field facilitators each day. The transcripts were read and checked independently by the local investigators for verification.

The data were analyzed through thematic analysis. Major themes were derived from the theoretical framework of the HBM. However, subthemes were induced from the text itself through repeated reading. After reading the transcripts, the investigators identified emergent themes, and then coded each theme to delineate individual topics identified during the discussions. Statements were grouped by code to the corresponding theme. Once themes were established, the transcripts were re-read to ensure the themes appropriately reflected the content of the data. Themes were then compared by sub-group: community leaders, women, and men’s FGD groups to identify similarities and differences.

The themes were presented to the field researchers in a two-day meeting, in order to validate their relevance and correct representation of the data. All themes identified were felt to capture the discussions from the FGDs.

The findings were presented in narratives by thematic areas using the HBM as the theoretical framework. The quotes included in the results were typical views expressed in each FGD to exemplify emergent themes.

### Ethics approval

Ethical approval was obtained from the Ethical Review Board of Jimma University and the PATH Ethics Committee. All study participants were given detailed information about the study using script approved by the ethics committees, provided verbal consent before participation,and were willing to participate in the study. Participants were provided with refreshments after the FGD.

## Results

Eighteen FGDs (six FGDs with men, six FGDs with women and six with community leaders) were conducted. Each FGD comprised of eight to twelve participants. For the FGDs with community leaders, religious leaders, community based insurance scheme (“Idir”) chairpersons, and other key community resource people, participants were purposely selected. A total of 168 participants (112 males and 56 females) were included. The age of the participants ranged from 21 to 70 years. All participants in the FGDs with community leaders were men, as women currently have limited participation in community leadership in Ethiopia. All participants engaged well with the topic and responded enthusiastically to the questions.

The findings are presented in four thematic groups: awareness of CC, perceived etiology of CC, perceived benefits of treatment, and perceived barriers to treatment. Across the data there emerged a strong interplay between the knowledge and attitudes about CC and the resultant health seeking behaviors. In general, the findings showed no major gender differences, nor differences between urban and rural participants, within the themes. The findings by sub-group have therefore been integrated within each theme, and presented as a whole. However, any specific differences identified between these sub-groups are highlighted in each theme, as appropriate.

### Awareness of CC

Most participants from Addis Ababa had heard of “cancer” but none spontaneously mentioned CC. In contrast, rural participants had limited awareness about any type of cancer. In particular, awareness about CC was almost non-existent.

Cancer is known in the local languages Afan Oromo and ‘Ahmaric as “tenecha” and “nekersa”, respectively. Addis Ababa participants further categorized cancer into two types, namely ‘Wondie’ -meaning masculine and ‘Setie’-meaning feminine types. According to them, the ‘Wondie’ type is fast growing and relatively easy to cure, and the ‘Setie’ type has a poor outcome. Although most of the participants from Addis Ababa had heard of “cancer”, they often did not know its symptoms and treatment options. They believed that cancer is a serious and often fatal disease. Approximately half of the participants from Addis Ababa thought that cancer was incurable. Men and community leaders had better awareness of cancer than women and this was mainly evident in Jimma.

In Addis Ababa, when participants were invited to list cancers that they had heard of, breast cancer was mentioned frequently; however cancer of the uterus and cervix were rarely or never mentioned. The uterus was understood to be a single organ with no distinction made between cancer of the uterus and cancer of the cervix. However, when the signs and symptoms of CC were explained to participants, the majority of participants from all study sites stated that the illness was common in their community and cited many examples of women who had died or were suffering from a similar illness.

### Perceived etiology of CC

The participants believed CC was due to a variety of socio-cultural and religious behaviors including promiscuity, a violation of normal sexual behaviors (frequent sexual intercourse, multiple sexual partners, early sexual intercourse), the devil’s intervention ‘ Likift’, wrath of “Attete” or “Wodaja”, failure to carry out proper rituals, evil spirits, exposure to the sun’s rays, urinating on dirty areas when it was sunny, ‘Mich’(sun allergy), poor personal hygiene, multiple pregnancies, abortion, and/or a mismatch in the size of genital organs of opposite sexes. However, none of the participants mentioned an infection/”germ” as the cause of CC. In explaining the association between CC and the devil’s intervention/God’s punishment a religious leader stated:

“…*Istihada, meaning punishment that occurs when the devil kicks a woman’s womb. This is an explanation from religious book.*”

Women from all urban areas commonly thought that lack of personal hygiene was a cause of CC. One of the participants said,

“…*First we females have to keep our personal hygiene by douching in the evening and in the morning… we have to clean out and clean our genitals, wash our underwear/ pants to prevent infections. …We also do not have to dry our underwear in the direct sunlight, and we do not have to hang it up on a fence. We have to keep somewhere inside the house to make it dry or bask it under a shadow*.”

Exposure to sunlight, and the effects of the sun or heat exposure were commonly thought to cause CC. Participants believe that either urinating facing the sun, or water vapor, coming from the ground produced by sunlight, enters the uterus during urination in an open space. Less commonly, CC was thought to be caused by sitting on a seat heated by the sun.

Some participants thought early age of marriage (which is common in Ethiopia) to be the cause of CC. One male participant said,

“…*As I heard about CC, when somebody has sexual intercourse with a girl who is kid or not matured, since her cervix may not open wide enough during delivery it could burst and get wounded and then cancer occurs.*”

Some participants from both Addis Ababa and Jimma thought CC to be due to trauma to the female genitalia from friction during coitus. Some believed that a large penis size causes more trauma and therefore increases the female sexual partner’s risk of developing CC.

### Perceived benefits of treatment

The majority of participants believed that modern medicine cannot cure CC, as the cause of the disease was due to supernatural powers, the devil, and/or punishment for violating normal sexual behaviors. As such, participants do not believe that modern medicine would be effective in such circumstances.

For example, a participant from Jimma stated that,

“…*this disease is not curable although people visit different treatment areas such as hospital .There is no perfect treatment, so the only option women have is death… .”*

Traditional medicine was preferred to modern medicine as it gives immediate symptomatic pain relief even though some participants thought that it is not curative.

One participant from Jimma said,

“…*they (women) prefer traditional healer to get fast relief and recovery from their problem and for the time being it gives some relief but it does not cure.”*

In contrast, very few participants from Jimma believed that early stage CC is curable by modern surgical or radiotherapy treatment.

Similarly, nearly half of the participants from Addis Ababa stated that the disease could be cured if treatment was given before reaching an advanced stage. Approximately half of the participants who thought CC could be cured believed that traditional medicine and holy water would be an effective treatment. One participant from Addis Ababa said:

“…*women who develop such a diseases go to ‘Tsebel’- meaning holy water*.”

The other half of participants believed that modern medicine would be the only effective treatment provided that early treatment is sought.

### Perceived barriers to treatment

Various factors including cultural, socio-economic, and beliefs about the disease and the health care system were found to affect the treatment seeking behavior for CC. Some of the barriers included: stigma associated with the disease, limited access to health services, the lack of awareness, and the asymptomatic nature of the disease. A major barrier to seeking treatment is the stigma and discrimination affected women experienced by their family and the community. As the community commonly believes the cause of CC is due to unacceptable social behaviors, women are therefore reluctant to disclose their condition due to the social consequences.

A community leader from rural Jimma stated,

“…many women rely on home based traditional treatment as they do not like to disclose the disease to the community owing to its perceived association the diseases with frequent sexual intercourse and multiple sexual partners.”

Stigma against the disease also plays a significant role in delaying health seeking for the disease from the modern medical institutions. One of the participants from Addis Ababa stated,

“…*people keep their disease secret, naming their illness as gastritis, kidney problem, and so on*.”

In addition, some participants from both Jimma and Addis Ababa explained that CC is cause of divorce as husbands do not want to live with a woman who has CC. A male participant from Jimma stated,

“… *men ignore their wives if they have symptoms of CC and the victim women themselves feel self enacted stigma and feel themselves as less important person and divorce will happen*”

Common barriers in seeking modern treatment for CC were related to the limitations in available and accessible health care services. Few diagnostic and treatment facilities are currently available in the country, and the few that are available are concentrated in Addis Ababa, discouraging the vast majority of the community from accessing care. Women living outside Addis Ababa must travel long and costly distances to receive diagnostic and therapeutic care.

One participant from rural Jimma said*,*

“…Women have to go to modern and expensive health facilities in Addis Ababa to get treatment. However, they cannot afford to go to Addis Ababa and most remain suffering from the diseases.”

Some of the study participants mentioned that the cost of treatment, which is only available in Addis Ababa, was not affordable for the majority of women, even for those living in Addis Ababa.

A participant from Addis Ababa stated,

“…*most women are not interested to visit the health facilities for their problems. The problem and the inconveniences within the health facilities to get diagnosed and treated discourage patients from visiting health facilities. It is clear that our health service delivery has a problem. Drugs are too expensive*.”

Low awareness amongst the participants about the existing services that are available exacerbated the perceived barriers. Most women were not aware of any available health services for CC.

### Health seeking behavior

Most of the participants emphasized that early treatment seeking was very limited, particularly in rural areas. They stated that most women with CC symptoms only seek treatment after the disease reached an advanced stage with the women suffering intolerable pain. A participant from Addis Ababa said,

“… in our community there is a habit of going to health institutions when it reaches a stage where they are unable to tolerate the pain.”

More than half of the participants from Addis Abba and a few from the urban Jimma area knew that treatment for CC was available in modern health facilities. There were two views regarding the preferred choice of treatment. Firstly, the majority of participants in Jimma believed that traditional healers are superior to modern medicine because they are easily accessible; modern medicine is not satisfactory, and the cost of treatment is more affordable. However, some participants did not support traditional remedies for the disease. They thought that traditional healers give herbs to their clients which further exacerbates the disease. Some knew of women who had used traditional medicine and subsequently died. A participant from urban Jimma explained,

“…There is a traditional healer in Jimma town to whom women go for treatment. The man gives the traditional medicines in the form of powder. Many died because of the failure of that medicine.”

In contrast, a few participants believed treatment for CC was available in the modern health institutions and that women attend to seek treatment. A major issue identified by some participants was that despite some women seeking modern medical care early, the disease is not diagnosed due to a lack of diagnostic capacity at the health facility, while almost all of the participants were not aware of the existence of screening facility at any level in the health care system. Additional health system factors included the lack of hospital beds to provide the necessary inpatient care.

Due to the aforementioned barriers to seeking modern health care, women often only seek health care in modern health facilities after exhausting the traditional remedies and/or holy water. In Addis Ababa, the majority of participants thought that about half of the women seek care from a traditional healer first, then seek modern medical care only after traditional remedies are ineffective. Those women who do seek treatment from the modern health system often present late in their illness, and then revert back to traditional remedies as modern health care is unable to provide services for such advanced disease.

A community leader from Addis Ababa stated,

“…*a woman who develops such a diseases first visits a traditional healer and then goes to health facilities after the disease progressed to severe stage*.”

Traditional medicines can be administered at home which is preferable due to cost and confidentiality.

A participant from Addis Ababa stated,

“…women prefer to take traditional means at home rather than visiting modern health facility. Only few individuals prefer modern treatment.”

However, in rural settings almost all women prefer the traditional remedies and are less likely to seek modern health care for such health conditions.

## Discussion

This study found that there was a strong interplay between the knowledge and attitudes of CC and the resultant health seeking behaviors. The community’s awareness of CC was low, but when the symptoms were explained, the participants recognized that the condition was common, with most participants being aware of women affected by this condition. Despite this, there were substantial psycho-social, socio-cultural, and health system barriers to effective health seeking behavior for women with CC. In this study, there was high level of perceived severity of CC in the community which could be attributed to both the high fatality rate of the disease, and the noticeable symptoms of late stage disease. As the disease is thought to be caused by breaching social taboos or undertaking unacceptable behaviors, modern treatments are thought to be ineffective in such cases. Due to such beliefs about the cause of CC, affected women are ostracized and suffer marked social stigma.

Our results also showed that misconceptions and poor awareness about the disease by its formal medical term were common amongst participants, which is consistent with the reports from other countries [11]. The concept of a communicable etiology was not reported in our study which is similar to findings from other studies [12,13]. This misconception may also result in poor health seeking behavior because of a false sense of not being vulnerable to CC provided acceptable social and cultural practices are adhered to. This finding is consistent with other studies [14-17]. According to the HBM, if individuals regard themselves as susceptible to a condition, believe that the condition would have potentially serious consequences and that a particular course of action (e.g., seeking early treatment) would be beneficial in reducing either their susceptibility to or severity of the condition, and believe the anticipated benefits of taking action outweigh the barriers to action, they are likely to perform the recommended action. Hence, acknowledgement of susceptibility and severity provide the motivation to act. The perception of benefits (provided there are no barriers) provides a preferred course of action (e.g., the preference of either traditional or modern remedies for CC) [9].

This study identified substantial barriers and challenges for early health seeking behaviors for CC. The identified barriers were related to the following four inter-related factors: the insidious nature of the disease, individual level factors, community level factors, and institutional factors. Our findings suggest that even when symptoms are present, health seeking is further delayed due to stigma based on local perceptions of the cause.

Individual level factors, including low awareness and misconceptions about the etiology of the disease, lead to seeking ineffective traditional remedies. The majority of the study participants were not aware about prevention of the disease including screening services for early detection and treatment of precancerous lesions. This finding is consistent with reports from other countries [11,18,19]. However, this is not surprising considering that screening and treatment services in Ethiopia are extremely limited and concentrated in urban settings. Beliefs concerning the cause of the disease are a crucial determinant of subsequent health seeking behavior. Affected women therefore tend to initially seek treatment from traditional healers and use holy water which is similar to other studies [16,20-24].

In this study community level factors were often found to determine individual behaviors. Marked social stigma and discrimination against affected women by the community force women to hide their condition and to avoid being exposed, as they will be accused of breaching social taboos or failing to adhere to sexual norms. This experience is therefore a major factor in discouraging women from seeking health care. Other studies reported that in order to avoid stigmatization, women avoid health care professionals, particularly in public settings [20,23,25,26]. In addition, these accusations by the community prevent affected women from receiving emotional support and result in unnecessary discrimination.

The health care system was found to be an important barrier to early treatment seeking behavior. Institutional level factors, including the lack of availability of appropriate services and their financial and logistical inaccessibility, deter women with CC from seeking health care. Effective health services need to be in place prior to raising awareness of the potential for preventing CC. These services are currently very limited in Ethiopia and for this reason alone, it is not surprising that modern medicine was not a preferred option for initial treatment. Moreover, there was a strong belief that diagnostic services and treatment of cancer were lacking in the Ethiopian health care system [25]. The functioning of the health services and economic factors on health-seeking behavior for CC were also found in other studies conducted in Africa [24,26]. This is consistent with other studies that have shown that availability and accessibility are critical for health care utilization [27].

Nevertheless, two main patterns of health seeking behavior were observed. First, most women prefer traditional remedies. Traditional healers were preferred as women believe they offer immediate symptomatic pain relief. Moreover, traditional remedies are more readily accessible than modern treatment and may be administered discretely without the need of disclosure to other family and community members. Other studies have found that when barriers are minimal, the likelihood of adopting the behaviors is increased [8,28].

Secondly, modern health care is often only sought after repeated failed attempts to cure the condition by traditional means. This practice was consistently reported amongst participants regardless of their social status.

An iterative pattern of health seeking characterized by switching between modern and traditional remedies observed in this study was also reported from other countries in East Africa [29]. This could be due to the low confidence in the treatment being effective in the modern health facilities due to aforementioned reasons.

The prevention of CC needs a number of factors to be effectively in place. The community needs to be aware of the etiology of CC, barriers to accessing health care need to be removed, the importance of screening asymptomatic women needs to be understood, accessible, and available, and treatment options need to be functional. This qualitative study identified that there were important factors and challenges preventing health care treatment for CC for Ethiopian women.

In conclusion, this study revealed various insights into the knowledge, beliefs, and practices of the community regarding CC. The obvious symptoms of CC have a profound negative impact on the social functioning, quality of life, and acceptability of affected women and create a reluctance to seek any form of treatment. Despite the awareness of the severity of CC, the misconceptions related to its etiology has a negative impact on the health seeking behavior and the receipt of any emotional support by affected women. Traditional remedies were the most preferred initial treatment for CC. However, the pattern of health seeking behavior alternated between traditional remedies and modern treatment methods as women presented for treatment at a late stage rendering any treatment ineffective. CC prevention programs must address psycho-social, cultural and religious barriers, and health service factors that influence appropriate health seeking behavior through appropriate behavioral change communication strategies.

## Competing interests

We declare that we have no competing interest.

## Authors’ contributions

Conceived and designed the study: AA, FMR, AD, ZB TB, HS, KM. Coordinated the field work: AA. Analyzed the data: AA, TB, ZB. Drafted the manuscript: ZB, TB and AA. Reviewed the article: AA, TB, AD, HS, KM, FMR. Critically reviewed several drafts of the article: VT. All authors have read and approved this manuscript.
